# Mechanical effects: challenges for high-field superconducting magnets

**DOI:** 10.1093/nsr/nwac220

**Published:** 2022-10-18

**Authors:** Xingyi Zhang, Jinggang Qin

**Affiliations:** Department of Mechanics and Engineering Sciences, College of Civil Engineering and Mechanics, Lanzhou University; Institute of Plasma Physics, Chinese Academy of Sciences

## Abstract

Due to its clean products and sufficient raw materials, fusion energy is expected to become one of the main solutions of the energy crisis and ensuring the sustainable development of human society, which is a long-term strategic frontier field. The promise of fusion energy is to constrain the motion of high-temperature plasma by the high magnetic field generated by superconducting magnets, and then achieve controllable thermonuclear fusion. Fusion power is proportional to the fourth power of the magnetic field strength. Thus, future commercial fusion reactors need a higher magnetic field as the basis for sustainable development [1].

In order to verify the scientific and technological feasibility of fusion energy, China, the United States, the European Union, Russia *et al*. have jointly participated in the construction of the International Thermonuclear Fusion Test Reactor (ITER), which is expected to produce the first plasma discharge by 2025 [2]. Currently, China is leading the world in many fields of fusion energy research. For example, the experimental advanced superconducting Tokamak (EAST) whole-superconducting Tokamak located at the Institute of Plasma Physics in the Chinese Academy of Sciences has achieved a repeatable world record of stable plasma operation at 120 million degrees Celsius for 101 seconds, which provides a solid foundation for ITER and also China's future Independent Building Fusion Reactor (https://www.cas.cn/syky/202105/t20210528_4790357.shtml). Prof. Jiangang Li, an academician of the Chinese Academy of Engineering, participated in and completed the design and construction of EAST plasma facing componments (PFCs) engineering by the support of the national ‘9th five-year plan’ major scientific and technological infrastructure, and presided over the completion of the national ‘11th five-year plan’ major scientific and technological infrastructure—EAST auxiliary heating system project. He also hosted the national ‘13th five-year plan’ major scientific and technological infrastructure—Integrated Research Facility for Critical Systems of fusion reactor comprehensive research facility for fusion technology (CRAFT). Many important scientific and technological problems have been solved and overcome by Prof. Li and his co-workers, which puts China's plasma physics research and fusion engineering technology at the forefront of global engineering.

## THE CHINESE FUSION ENGINEERING TESTING REACTOR

**NSR**: Can you give a brief introduction to the basic status of the Chinese Fusion Engineering Testing Reactor (CFETR) and the preparation works currently in progress?

**Li**: CFETR is a major project designed and developed by China independently and with international cooperation to carry out next-generation fusion reactor research. The CFETR engineering design was supported by the national Special Project for Magnetic Confinement Fusion Energy Research, and started in 2015. The engineering design of the main components was completed in 2019 [3]. Since then, some core components and key materials are being produced. For example, high-performance Nb_3_Sn superconducting materials have been mass produced with Jc∼2300A/mm^2^. By undertaking tasks about ITER PF6, CFETR central solenoid model coil (CSMC) research and CFETR toroidal field (TF) prototype magnet, CFETR magnet technology has made great progress [4].

**NSR:** The core component of a fusion device is a superconducting magnet. What are the differences between the CFETR and ITER superconducting magnets?

**Li:** Compared with ITER magnets, CFETR superconducting magnets are more difficult [5]:

- Magnetic field parameters: the maximum field of an axial field coil is 14.5 T (that of ITER is 11.6 T), the maximum current
  CFETR is a major project designed and developed by China independently and with international cooperation to carry out next-generation fusion reactor research.
  —Jiangang Li
  is 100 kA (68 kA), and the energy storage is ∼157 GJ (50 GJ);
- Magnet material: high-field Nb_3_Sn and high-temperature superconducting material will be first used in the axial field coil and central solenoid coil, respectively, while ITER does not use these materials;
- Magnet stress: the maximum stress of the axial field coil will be increased by ∼50%, which significantly increases the requirements for magnet structure built with high-strength materials.

**Figure 1:**
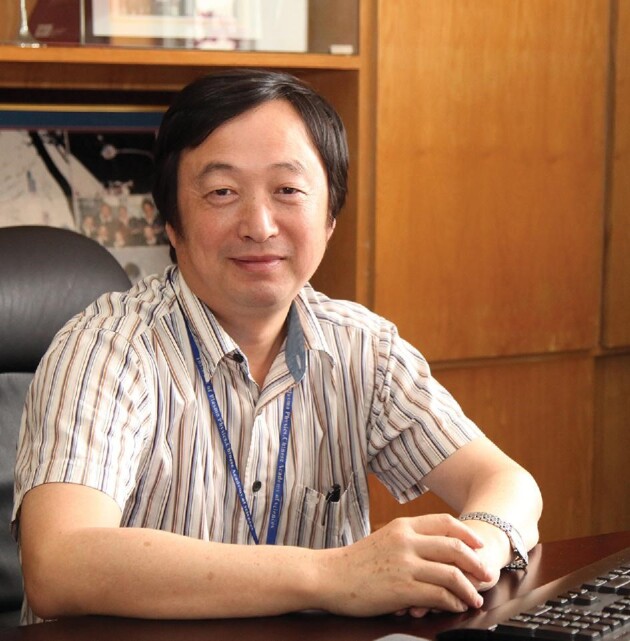
Jiangang Li of the Institute of Plasma Physics, Chinese Academy of Sciences *(courtesy of Jiangang Li).*

## BASIC MECHANICAL PROBLEMS

**NSR:** The safety and stability of superconducting magnets depend on the basic superconducting materials and conductors. What difficulties does our country have in the development or selection of superconducting materials and conductors at present, and how do we solve these difficulties?

**Li:** The superconducting magnets of the future fusion reactor are characterized by a strong magnetic field and a large current. Therefore, high stress is one of the key problems faced by fusion magnets, and has an effect on the stability of the current-carrying performance of the magnets. Whether it is Nb_3_Sn-based superconducting material or high-temperature superconducting material, the critical current shows significant stress sensitivity [6]. Recently, Zhou *et al*. [7] reported a new phenomenon of mechanical perturbation-induced quench. Jing *et al.* numerically simulated the thermal-magnetic-mechanical properties in bulk and films. Their results enhanced researchers’ understanding of the relationship between cracks and magnetic flux motion [8,9]. Therefore, it is necessary to focus on studying stress distribution in the design of high-field superconducting magnets (including superconducting conductors) in order to reduce or avoid their performance degradation through optimized structure design. For example, short-pitch (STP) cable in conduit conductors (CICC) exhibit excellent characteristics of inhibiting shunt temperature degradation under cyclic electromagnetic loads. This conclusion has been validated by SULTAN’s experiment [10] and the theoretical prediction given by Lanzhou University [11]. Therefore, STP-type conductors will also become the first choice for national CFETR high-field coils.

**NSR:** The study of the mechanical effect of superconducting materials includes many basic experimental measurements. Has there been any important progress in the basic experimental measurements of superconducting materials?

**Li:** The main basic experiments of superconducting materials involve electromagnetism, mechanics, thermodynamics and other areas in physics. In terms of electromagnetism, the Institute of Plasma Physics has a 14 T background-field experimental condition, which can realize the performance evaluation of superconducting materials under the simultaneous loading of electricity, magnetism and heat. The results have been recognized by internationally renowned laboratories. In terms of mechanical measurements, the research group from Lanzhou University has great experimental facilities, representing the highest level of mechanical property evaluation of superconducting materials in China. In response to high-field demand in the future, Lanzhou University together with the Institute of Plasma Physics and the Institute of Modern Physics are planning to build a superconducting mechanical experimental measurement platform with a magnetic field of 15 T. Meanwhile, a national facility called CRAFT will have several high field magnets up to 16 T with large sizes.

## MECHANICAL EFFECTS IN THE FUTURE

**NSR:** In early designs of superconducting magnets, mechanical response was not considered. Now, due to the increase of magnetic field strength, the mechanical response is more significant. Do you have any opinions and suggestions for the future design of superconducting magnets, especially high-field superconducting magnets?

**Li:** The main challenge of high-field magnets in the future comes from mechanical responses related to superconductivity, which has become the consensus of international and domestic researchers. For the design of high-field magnets in the future, it is first necessary to develop new materials that resist strong stress. Secondly, in the design of magnet structure, it is necessary to break away from traditions and develop a new magnet structure. Finally, at the stage of preparation, engineering feasibility must be ensured, and multi-directional testing needs to be carried out. In addition, in terms of theoretical predictions, the main requirement is to establish an electromagnetic-thermal-mechanical coupling three-dimensional computational model.

The main challenge of high-field magnets in the future comes from mechanical responses related to superconductivity.

—Jiangang Li

Prof. Youhe Zhou, an academician of the Chinese Academy of Sciences, coming from Lanzhou University, has achieved some substantial progress in this research direction [12]. It is hoped that the domestic research institutes mainly engaged in superconducting magnets will cooperate with Lanzhou University to jointly improve the research ability of superconducting magnets in China.
